# BioMGCore: A toolkit for detection of biological metabolites in microbiome

**DOI:** 10.1002/imo2.70036

**Published:** 2025-06-22

**Authors:** Guang Yang, Guohao Yu, Qian Cui, Xueping Huang, Kexin Guo, Shulei Jia

**Affiliations:** ^1^ Jiangsu Key Laboratory of Marine Bioresources and Environment, School of Ocean Food and Biological Engineering, Co‐Innovation Center of Jiangsu Marine Bio‐industry Technology, Jiangsu Key Laboratory of Marine Biotechnology, Jiangsu Marine Resources Development Research Institute Jiangsu Ocean University Lianyungang China; ^2^ School of Basic Medical Sciences Tianjin Medical University Tianjin China

## Abstract

The keyword matching and gene proximity principles were used to accurately identify core gene clusters in microbial genomes. The metabolic gene clusters can be classified into different taxonomic groups according to Domain, Phylum, Class, Order, Family, Genus, and Species. BioMGCore can achieve batch statistics for secondary metabolites prediction.

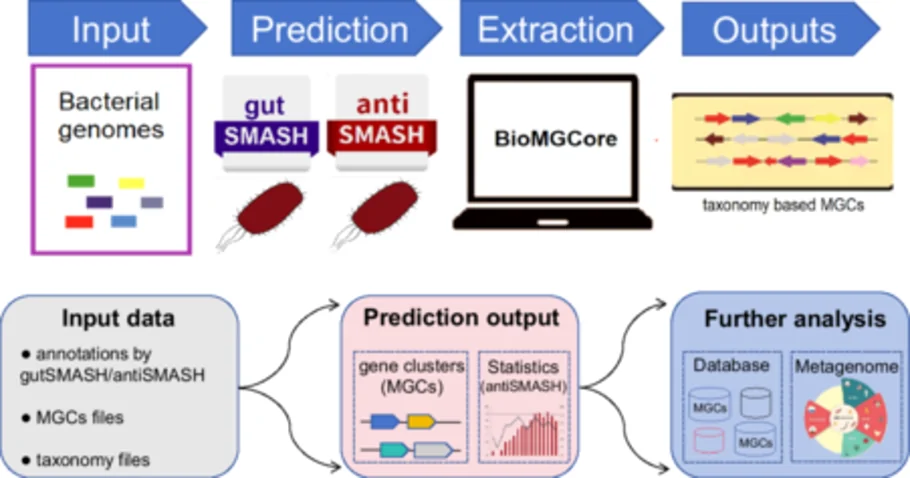

## CONFLICT OF INTEREST STATEMENT

The authors declare no conflicts of interest.

## ETHICS STATEMENT

No animals or humans were involved in this study.

To the editor,

Microorganisms, including bacteria and fungi, are ubiquitous in nature and have been associated with food systems for centuries [1, 2]. The gut microbiota can also utilize small‐molecule metabolites to synthesize extracellular polysaccharides, which are crucial for bacterial biofilm formation and the stability of gut microbiota [3, 4, 5, 6, 7]. In addition to the primary metabolites of microorganisms, the microbial secondary metabolites provide enormous potential for the development of new drugs. The microbial metabolites are encoded and catalyzed by the core metabolic gene clusters (MGCs), whose genes are physically located on the chromosomes. Accurately identifying these functional MGCs in microorganisms is of great significance for drug synthesis, disease diagnosis, and human health. To identify and extract the MGCs from complex microbiota, the best choice is to make an analysis based on both gutSMASH and antiSMASH with Hidden Markov Models [8]. However, currently, the results can only be visualized and manually processed through interactive modes on web pages. Users can neither batch process gene sequences, nor obtain the species origins of these MGCs, which is not conducive to large‐scale genome analysis. Therefore, to resolve the massive data processing problem, we developed the keyword matching and gene proximity‐based algorithm [9] to quickly identify and extract intact MGCs instead of massive noncritical functional genes extraction. It can achieve data dimensionality reduction by extracting core functional gene clusters from a large number of annotations by gutSMASH or antiSMASH. The developed toolkit can help to explore the primary and secondary metabolites from microorganisms, which helps to improve analysis efficiency and explore potential microbial metabolites.

## 1 RESULTS AND DISCUSSION

### Implementation and performance assessment

**Figure 1:**
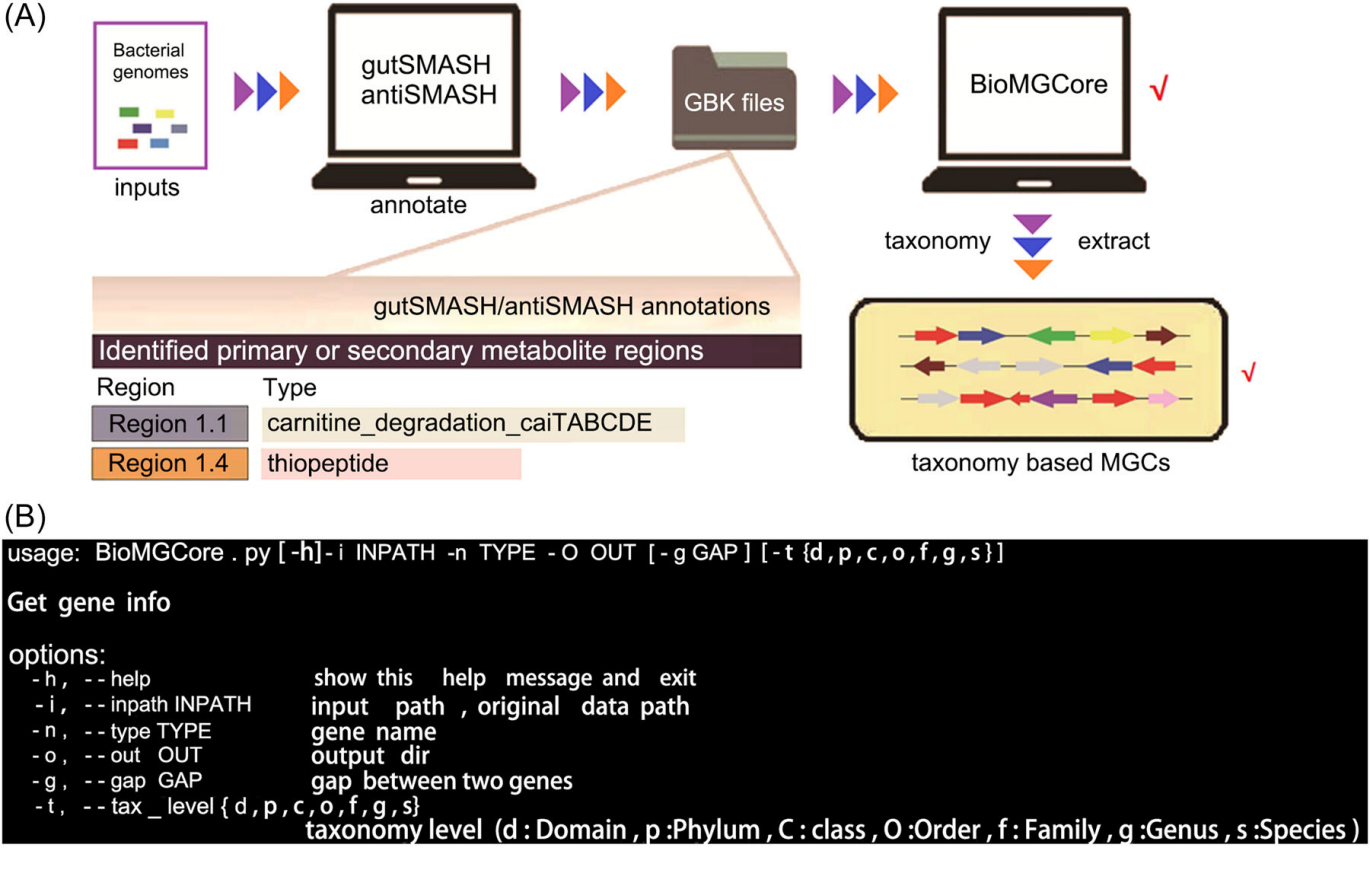
Pipeline and usage of BioMGCore. (A) Pipeline of the BioMGCore. The bacterial genomes should be annotated by gutSMASH or antiSMASH. Then, the program could identify and output the taxonomy‐based core MGCs through built‐in logic. Both cultivable bacterial genomes and the assembled species‐level genome bins (SGBs) are suggested for analysis. (B) Usage of the toolkit with description of options and default parameters. The main program can extract specified MGCs and classify them according to Domain, Phylum, Class, Order, Family, Genus, and Species.

Before starting sequence extraction of the functional MGCs, the genomic annotation should be conducted by antiSMASH or gutSMASH (Figure 1A), and the generated files should be renamed with the format like “results_genome_file_name.” Notably, the folders named species and gene_label2 must be placed in the same directory with the annotated results (https://github.com/xielisos567/BioMGCore/tree/main/examples). When the “‐n” option is enabled, the application scans for specific types of MGCs in the HTML files produced by antiSMASH or gutSMASH, as illustrated in Figure 1A. The core genes were extracted directly from the GenBank flat file (GBK), which provided validated cluster boundaries and avoided arbitrary cutoffs. If genes within the gene cluster are separated by any other unrelated genes, then the “‐g” parameter can set the number of unrelated genes (genes with intervals). All genomes exhibiting ≥2 genes in synteny (defined as being separated by ≤10 genes based on locus tag) could be selected as candidates [10]. In this case, we can accurately extract the intact neighboring MGCs. In addition, we introduced a “‐tax_level” parameter to allow users to select their desired taxonomic rank for analysis, which generated summary statistics for MGC distributions at different taxonomic levels. The taxonomy file format was standardized with normal form or the 7‐level system (Domain, Phylum, Class, Order, Family, Genus, and Species), which was practical for user‐assembled genomes like metagenomic‐assembled genomes (MAGs). Option “‐o” will output the extracted results according to the MGC type entered by users. For example, command‐line usage of BioMGCore is shown as below: *python3 BioMGCore.py [‐options]/{‐n* < *MGC type* > *‐i <directory*> *‐o <outputs*>*}* (Figure 1B). The Pandas module serves as a crucial tool for data mining and data analysis and is constructed based on Numpy [11].

### Workflow and step‐by‐step usage of BioMGCore

The γ‐butyrobetaine (γBB) gene cluster *caiTABCDE* of *Escherichia fergusonii* ATCC 35469 (accession: GCF_000026225.1) was searched from the visual interactive web page of gutSMASH. Each brown colored gene represents the core gene of the MGC. Each gene contains a protein ID and location information, with the locus tag being consecutive with each other. The main steps for this process are as follows: According to the designated type of MGCs entered by users, BioMGCore firstly accesses the “HTML” file to confirm the MGC category and its region (step 1 and step 2). Then, the toolkit scans the MGCs with detailed genes such as *caiTABCDE* in the “gene_label2” folder, and then searches in the “GBK” file to extract the corresponding genes. During this process, the program will execute neighboring gene judgment logic to accurately extract core genes based on the characteristics of MGCs (step 3 and step 4). Finally, the BioMGCore toolkit scans the species information file corresponding to the genomes in the “species” folder and extracts the species information. Then, the results generated from the above steps were merged to form the species‐specific nucleotide and amino acid sequences, respectively (step 5).

**Figure 2:**
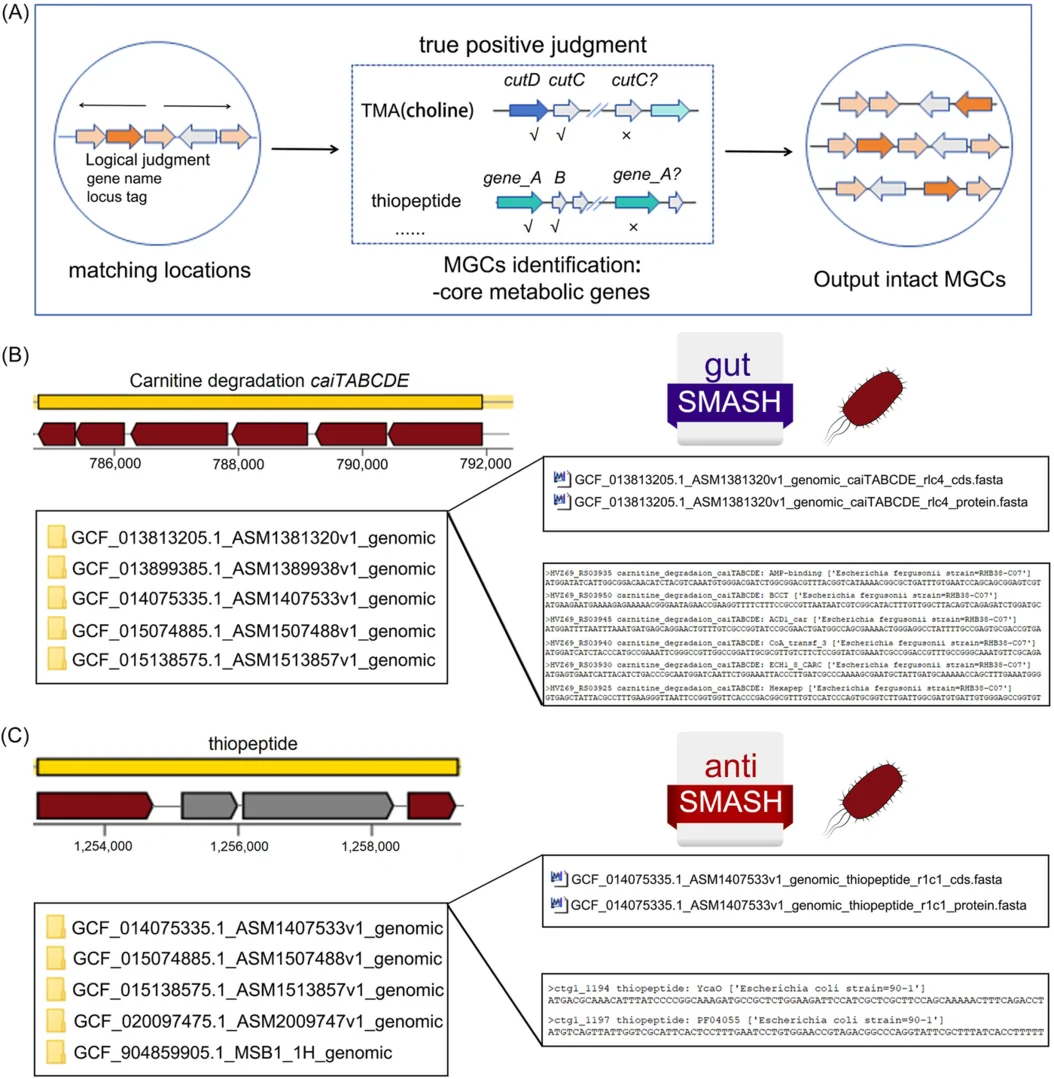
The working logic and examples of BioMGCore extraction. (A) The logic of metabolic gene clusters (MGCs) identification. The keyword matching and gene proximity principles are used for the identification and extraction of core MGCs. (B) Example outputs of BioMGCore for gutSMASH. (C) Example outputs of BioMGCore for antiSMASH. The extracted results contained complete core MGCs with taxonomic information. The extraction for each annotated genome not only included nucleotide sequences but also amino acid sequences.

Furthermore, five reference bacterial genomes were chosen and annotated through gutSMASH with default parameters. Then the annotated results were further processed with the BioMGCore toolkit. After putting the species information of each genome and the gene information to be extracted into the species folder and the gene_label2 folder, respectively, then the extraction process was conducted as follows: *python3 BioMGCore.py [‐options]/{‐i <directory*> *‐n caiTABCDE ‐o results}*. Firstly, the toolkit scans and matches the annotation results based on gene names, locking the corresponding gene tags. Then, the automatic decision‐making could classify the true positives and false positives based on the characteristics of MGCs. As the genes within gene clusters are located closely with each other, the genes with far distance, although their gene names match the extraction criteria, will be judged as false positives due to the nonconforming gene interval. Only the results that meet the required gene interval will be retained, which greatly ensures the accuracy of the extracted gene clusters. That is the reason why suitable gene interval should be set based on the gene tags in the toolkit for MGCs detection (Figure 2A). Here, we strictly set the gene intervals with less than five [10], and finally, there generated five results containing intact MGCs with detailed species information. The extracted results were in consistent with the gutSMASH annotation on the website, indicating a high accuracy of the BioMGCore toolkit. The extracted results included the nucleotide and amino acid sequences of gene cluster, and the locus tag of each gene were consecutive with each other. For example, the locus tags of the *caiTABCDE* genes in *Escherichia fergusonii* strain RHB38‐C07 (GCF_013813205.1) are labeled from HVZ69_RS03925 to HVZ69_RS03950 (Figure 2B). Similarly, another five bacterial genomes were annotated by antiSMASH, and we extracted the results using the BioMGCore toolkit. The results showed that each genome contained an intact gene cluster of thiopeptide with two core genes arranging neighborly. For example, the gene cluster for thiopeptide synthesis could be extracted accurately from the genome of *E. coli* strain 90‐1 (GCF_014075335.1) (Figure 2C).

### Applications of BioMGCore in microbiome

To investigate the applications of BioMGCore in different environmental microbial communities, we further tested this toolkit. Through analysis of the 1520 genomes, it was found that at least 1266 bacteria had the ability to synthesize secondary metabolites, as the intact MGCs were detected in their genomes (Table S1). Among these secondary metabolite gene clusters, the functional MGCs for synthesis of cyclic‐lactone‐autoinducer accounted for the highest proportion (31.22%) among all the MGCs, and they coexisted with other secondary metabolite gene clusters. In addition, to classify bacterial members of the l‐carnitine→γBB pathway for trimethylamine (TMA) precursor synthesis, we analyzed the cardiovascular disease (CVD) associated metabolites in 25,755 reference bacterial genomes (March 10th, 2022) with BioMGCore. Through a multi‐step process involving the atherogenic intermediate, l‐carnitine, a nutrient rich in red meat, speeds up atherosclerosis by forming TMA and trimethylamine N‐oxide (TMAO) in a gut microbiota‐dependent manner [12], involving enzymes encoded by the *caiTABCDE* gene cluster. The sequence of the *cai* gene encoding the carnitine pathway in *Escherichia coli* has been determined [12, 13, 14]. Due to the presence of numerous genes with incomplete or poorly sequenced data in the NCBI database, the extracted genes must undergo further filtering to obtain complete reference gene sequences for subsequent analysis. For example, the reference length of l‐carnitine CoA‐transferase (K08298) was 405 amino acids; hence, the protein sequence with coverage less than 80% would be filtered, which was a domain‐based validation method for biological accuracy [15]. The filtered genes were subsequently merged with the compilation file for the next step. The combined file underwent de‐redundancy at 100% identity, resulting in a nonredundant gene data set for further analysis. Finally, the results showed that a total of 3541 bacterial strains contained intact *caiTABCDE* gene clusters, and most of them were *E. coli* and *Salmonella enterica*, both of which belonged to the members of Firmicutes (Supporting Information S1: Table S2). We constructed a species‐specific database of the *caiTABCDE* gene clusters, which can be conducive to the target treatment of CVDs (Supporting Information S1: Table S2).

## 2 CONCLUSION

In this study, we developed a MGC toolkit for analysis of biological metabolites. As an auxiliary toolkit, BioMGCore not only helps to extract core MGCs but also enables species‐level analysis. On the basis of the existing software, it can predict a series of known and inferred MGCs related to microbial functional analysis. It can be used to analyze the vast majority of microbial communities, including the human gut or environmental microbiota, and has been proven to be fast and accurate in genomic analysis. We believe that this study offers the community a valuable resource in conjunction with antiSMASH and gutSMASH for profiling species‐specific MGCs in microbial analysis. Moving forward, BioMGCore will continue to evolve by integrating emerging tools and methodologies, ensuring it remains at the forefront of microbial metabolites research.

## 3 METHODS

### Installation of antiSMASH and gutSMASH

Before executing the code, the microbial secondary metabolism and primary metabolite prediction tools should be installed and used. The antiSMASH could be downloaded on the online website https://dl.secondarymetabolites.org/releases/8.0.1/. Meanwhile, gutSMASH is a Python‐based pipeline developed from the antiSMASH 5.0 source code. The latest command‐line version is available and can be freely downloaded from GitHub https://github.com/victoriapascal/gutsmash/tree/gutsmash.

### Construction of the nonredundant gene dataset

To eliminate the redundant genes at 100% sequence identity, the CD‐HIT v4.8.1 software [16] was selected and set up. The parameters were configured as follows: ‐c 1 ‐n 5 ‐M 1600 ‐d 0 ‐t 8. The detailed explanation of the parameters is as follows: “‐c” means sequence identity, “‐n” means the word size selected for sequence alignment between pairwise sequences, “‐M” means Random‐Access Memory (RAM), “‐d” means using the field before the first space in the FASTA title as the sequence name, “‐t” means number of threads.

### Genomes and the appended documents

A set of genomes as inputs in FASTA or GenBank format should be prepared. The folder named “species,” containing the tab‐separated assembly summary file, is also required. For example, the reference bacterial assembly summary files can be downloaded from the NCBI RefSeq (https://ftp.ncbi.nlm.nih.gov/genomes/refseq), following the format normalization as described above. Finally, users should prepare a folder named “gene_label2,” and it contains the files with MGCs names, in which the detailed genes of MGCs could be checked. We have provided the regular genomic information downloaded from NCBI RefSeq (April 22th, 2025) and the detailed names of the gene clusters placed in the “species” and “gene_label2” folders, respectively. In personalized operation, users can check the genes from the “index.HTML” files generated by antiSMASH or gutSMASH. The labels of different MGCs are listed in Tables S3 and S4.

### Applications of BioMGCore in gut microbiota

The 1520 reference genomes from cultivated human gut bacteria were downloaded from the NCBI BioProject (PRJNA482748, https://www.ncbi.nlm.nih.gov/bioproject/?term=PRJNA482748) [17]. The genomes from human gut were annotated through gutSMASH or antiSMASH with default parameters, respectively. Then the annotations were analyzed through the BioMGCore toolkit. The statistical analysis was conducted with GraphPad v8.0.2.

## AUTHOR CONTRIBUTIONS

**Guang Yang**: Investigation; writing—review and editing; writing—original draft; data curation; funding acquisition. **Guohao Yu**: Data curation. **Qian Cui**: Software. **Xueping Huang**: Formal analysis. **Kexin Guo**: Data curation. **Shulei Jia**: Conceptualization; methodology; writing—review and editing; supervision.

## Supporting information


**Table S1.** Statistics of the antiSMASH annotations by antiSTAT.
**Table S2.** The γ‐butyrobetaine (γBB) database through BioMGCore.
**Table S3.** The labels of different MGCs in the database of gutSMASH.
**Table S4.** The labels and types of different MGCs in the database of antiSMASH.

## Data Availability

The data and scripts used are saved in GitHub (https://github.com/xielisos567/BioMGCore), along with comprehensive tutorials and example work‐flows. Supplementary materials (tables, graphical abstract, slides, videos, Chinese translated version, and update materials) may be found in the online DOI or iMetaOmics http://www.imeta.science/imetaomics/. The data that supports the findings of this study are available in the supplementary material of this article.
